# A novel underwater dam crack detection and classification approach based on sonar images

**DOI:** 10.1371/journal.pone.0179627

**Published:** 2017-06-22

**Authors:** Pengfei Shi, Xinnan Fan, Jianjun Ni, Zubair Khan, Min Li

**Affiliations:** College of IOT Engineering, Hohai University, Changzhou, Jiangsu, China; University of Glasgow, UNITED KINGDOM

## Abstract

Underwater dam crack detection and classification based on sonar images is a challenging task because underwater environments are complex and because cracks are quite random and diverse in nature. Furthermore, obtainable sonar images are of low resolution. To address these problems, a novel underwater dam crack detection and classification approach based on sonar imagery is proposed. First, the sonar images are divided into image blocks. Second, a clustering analysis of a 3-D feature space is used to obtain the crack fragments. Third, the crack fragments are connected using an improved tensor voting method. Fourth, a minimum spanning tree is used to obtain the crack curve. Finally, an improved evidence theory combined with fuzzy rule reasoning is proposed to classify the cracks. Experimental results show that the proposed approach is able to detect underwater dam cracks and classify them accurately and effectively under complex underwater environments.

## Introduction

Numerous factors such as cracks, abrasions, cavitation, and erosion can threaten the safety of a dam [1]. Out of these, cracks represent the primary danger because they can exist not only at the dam’s surface but also extend into the interior [2]. In other words, cracks in dams are the equivalent of mutations as dams accumulate internal damage [3]. Thus, cracks are always used to indicate the degree of risk in the field of dam damage, which has attracted the attention of numerous scholars [4].

Various traditional methods such as electrical prospecting, elastic wave testing, tomography, and ground penetrating radar [5–7] are employed to detect cracks in dams. However, some of these methods are expensive, and others are neither sufficiently convenient nor reliable. Recently, detecting underwater dam cracks using sonar images has become one of the most important methods because it is nondestructive, intuitive, convenient and efficient [8].

Sonar data is obtained based on echo intensity when the sonar beam scans the crack area. And the echo intensity is displayed on the sonar image screen using different gray levels. The gray levels in these sonar images represent information that can accurately reflect crack depth. However, the sonar images can not accurately reflect the crack features on the dam surface, since their echo intensities are always the same. Thus, the sonar systems used in practice always provide only low-resolution imagery [9]. In addition, underwater environments are complex, vary over time, and are susceptible to substantial interference [10–12], which leads to measurement signals being overcome by noise. Moreover, unstructured cracks are random and diverse, which makes them difficult to describe. Finally, the images obtained from sonar lack calibration, and features obtained from a sample image without manual review cannot accurately reflect the relationship between a crack in the image and an actual crack. As a result, sonar images of dam cracks are highly uncertain and fuzzy, making detection and classification difficult.

Many crack detection algorithms based on imagery such as neural networks, genetic algorithms, mathematical morphology and tensor voting methods, have been proposed [13–16]. Chen et al. [17] presented an adaptive underwater dam crack edge detection algorithm based on multi-structure and multi-scale elements. Kabir et al. [18] evaluated various edge-detection algorithms and noted that the statistics-based approach was the most efficient technique for damage assessment. Bernstone and Heyden [19] proposed a digital image analysis technique for crack monitoring using a standard webcam to acquire continuous data sets from concrete dams. Xu and Zhang [20] suggested an integrated model using digital image processing to develop a numerical representation of concrete structure defects.

The characteristic based detection methods mentioned above are always subject to substantial noise, thereby leading to low detection rate and high false alarm rate [13, 21]. In particular, when the interference exhibits the same characteristics as the target, it will make the detection more difficult. In addition, the methods mentioned above are focused on de-noising and edge detection. Clustering and region growing methods have also been used [22], but apparently not for underwater sonar images. Moreover, for the underwater sonar images, few methods jointly consider the crack detection and classification, which is common in the optical images of pavements [23]. In sonar images, fuzziness and uncertainty must also be taken into account in making a correct classification.

In this paper, a novel detection and classification approach for underwater dam cracks based on dual-frequency sonar images is proposed. Images obtained from DIDSON are used to conduct evidence fusion for classification purpose. Both frequencies are used in this paper as source evidence. These two types of source evidence are fused to perform classification. In this paper, two main tasks are considered together: underwater dam crack detection and classification. This paper proposes an improved crack detection algorithm based on clustering analysis and tensor voting. And then, with the results of the crack detection, an improved evidence theory combined with fuzzy rule reasoning is put forward to distinguish different types of cracks. In the proposed method, fuzzy evidence is used to reflect the fuzzy information in the images, and the uncertainty is decreased via evidence fusion.

**Fig 1:**
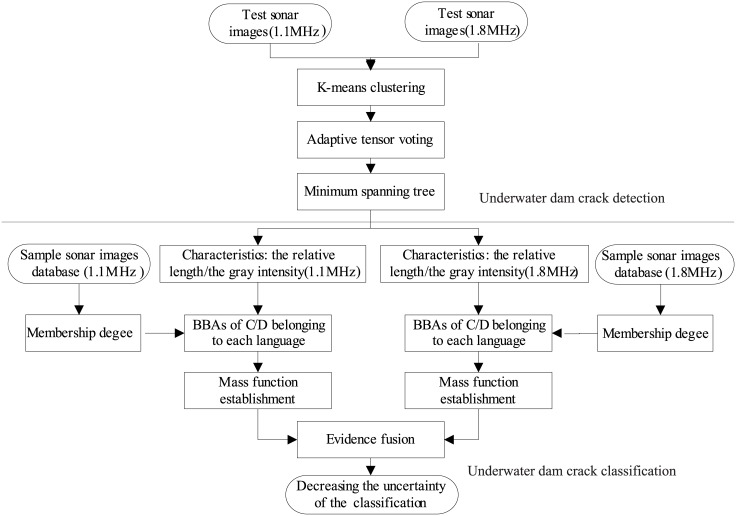
The workflow of the proposed approach.

To perform these two tasks, the classification scheme relies on following two characteristics of the target cracks: 1) the image regions are darker than their surroundings, and 2) the connected domain of the crack region is thinner than that of other regions. A workflow for the proposed approach is shown in Fig 1. Two main tasks are considered together: underwater dam crack detection and classification. First of all, the sonar images are obtained from the DIDSON with two different frequencies. And then an improved crack detection algorithm based on clustering analysis and tensor voting is proposed to detect underwater dam cracks. The detailed detection process is as follows. First, the sonar images are divided into image blocks. Second, a clustering analysis of a 3-D feature space is used to obtain the crack fragments. Third, the crack fragments are connected using an improved tensor voting method. Fourth, a minimum spanning tree is used to obtain the cracks. After obtaining the cracks, an improved evidence theory combined with fuzzy rule reasoning is proposed to classify the cracks. The detailed classification process is as follows. First, the characteristics of the crack regions are calculated to obtain the basic belief assignments (BBAs) based on the likelihood measure. Second, the BBAs of the characteristics are combined to classify the cracks based on the fuzzy rules and edge random set’s expansion guidelines. And the BBAs for the two sonar frequencies from different perspectives can be obtained in the same way. Third, in order to reduce the uncertainty of the classification and improve the robustness of the decision making, BBAs from the two sonar frequencies and different perspectives are combined based on the conditional masses. The proposed method will be introduced in detail in the next sections.

The main contributions of this paper are: 1) A detection and classification approach based on sonar images for underwater dam crack is presented. 2) In the proposed approach, both the local and global features are combined and used with block clustering and statistical analysis techniques. 3) The crack information gained from the sonar images, which contain substantial uncertainty, is mapped to a basic belief assignment. 4) The uncertainty of classification is decreased by updating the evidence, and the update rule is improved.

The next section illustrates the basic idea of the proposed approach. Some experiments are performed in the Experiments and Results section. A comparison with other methods proves the efficiency of the proposed approach. The last section concludes the paper.

## Materials and methods

### Sonar images

**Fig 2:**
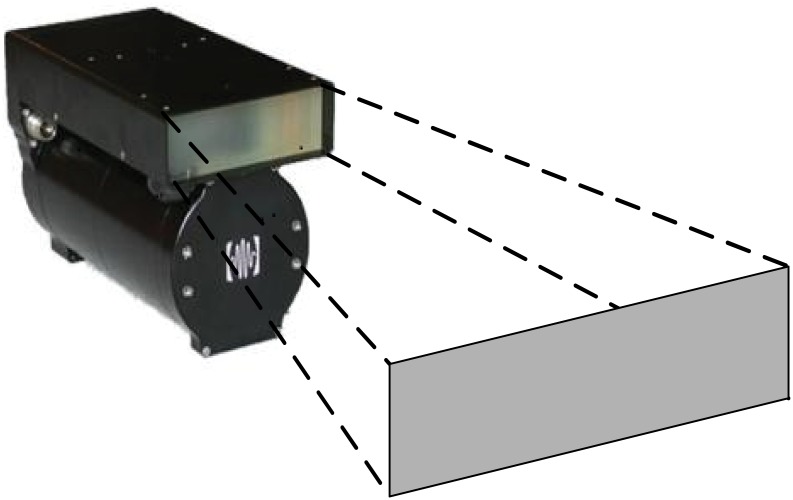
The model of DIDSON sonar.

In this paper, we obtained statistical crack detection results using twenty sonar images taken from a hydropower project, which were used as the sample database and the test database. All the sonar images were obtained using the DIDSON sonar from Dam of Longyangxia Hydropower Station by the author’s research team. DIDSON is a dual frequency identification sonar with operating frequencies of 1.1 MHz/1.8 MHz. The model of DIDSON sonar is shown in Fig 2. Detailed information concerning DIDSON can be found in [24]. When the images were obtained, the sonar system was perpendicular to the face of the dam which was more than 30 yeas old with cracks and other defects on it. The distance from the sonar to the defects was 500 cm, and the depth from sonar to the surface of the water was about 10 m. The images obtained from sonar were part of the dam and the resolution of the sonar images was 360 x 144 pixels. The experiments coded in Matlab 2011 were conducted on a PC with a 2.6 GHz CPU and 4 GB of RAM.

### Underwater dam crack detection

**Fig 3:**
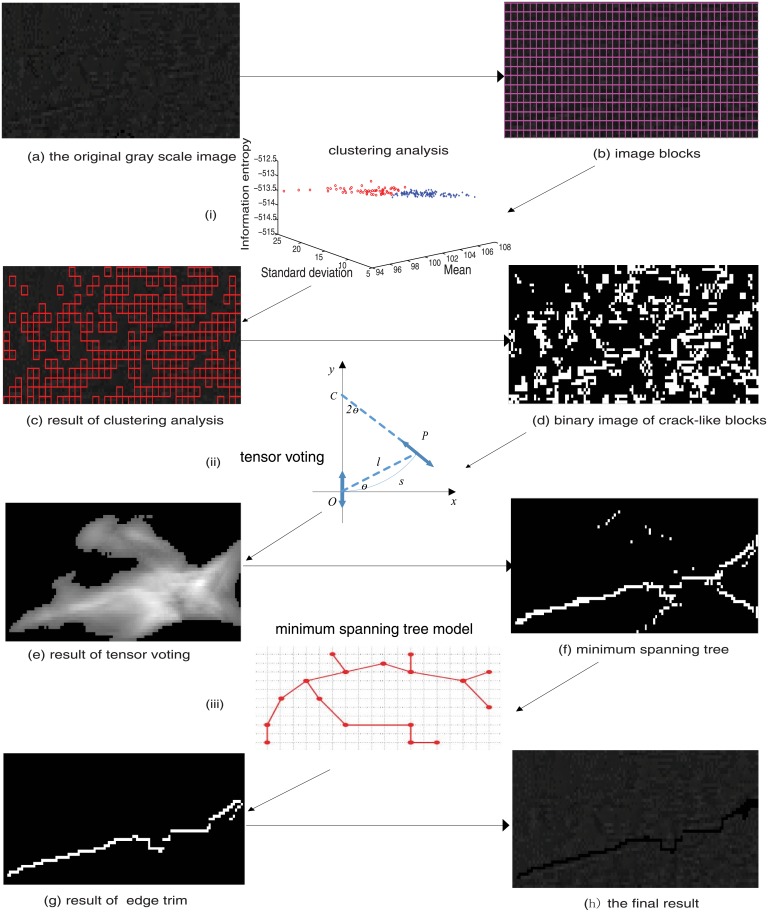
Flow chart of the proposed method. (i) clustering analysis, (ii) adaptive tensor voting, (iii) minimum spanning tree construction and edge pruning.

The proposed underwater dam crack detection method mainly consists of two steps: 1) Clustering analysis of image blocks. 2) Adaptive tensor voting of the crack fragments. The details of the proposed method is fully represented in the flow chart in Fig 3.

#### A. Clustering analysis of image blocks

First, the image matrix’s rows and columns are divided by a fixed value. Then, the image is divided into non-overlapping blocks. The block size should be chosen properly. When the blocks are too small, the number of false positive crack detections will tend to increase. In contrast, when the blocks are too large, the computation of the statistical features of tiny cracks will tend to vanish. The selection of the block size which will affect the detection results obviously is a limitation of this algorithm. In this paper, the blocks are 9 × 9 pixels, which represent a good trade-off between computational performance and crack detection accuracy. Three characteristic values are calculated for each block, which construct three feature matrix for the image: 1) the mean value matrix (Mm), 2) the standard deviation matrix (STDm), and 3) the information entropy matrix (IFE). A clustering analysis method is used to distinguish cracks from the background using 3-D spatial classifiers; each point identifies one image block. Then, the feature set in the 3-D space is defined as follows:

$$\begin{array}{c} F_{2}=\left\{(V_{1},y_{1})...(V_{n},y_{n}):V_{i}\in R^{3};y_{j}\in \left\{c_{1},c_{2}\right\}\right\} \end{array}$$ (1)

where *n* is the number of block points for the pattern vector *V*, which is constructed using Mm, STDm and IFE, and *y*_*i*_ corresponds to the *i*th block. The blocks are divided into two classes, namely, *c*_1_ and *c*_2_, denoting blocks containing non-crack information and blocks containing crack information, respectively. Furthermore, the K-means classification approach is used to facilitate the clustering analysis [23]. The clustering result in the 3-D feature space is shown in Fig 3(i). The classes that are labeled with red circles in the 3-D feature space belong to the target class *c*_2_, and the rest belong to the target class *c*_1_. After removing those blocks that are confirmed to have non-crack information, the remaining blocks are binarized by the Otsu method [25]. The binary image which contained crack segments and noise is shown in Fig 3(d).

#### B. Adaptive tensor voting of the crack fragments

Following the cluster analysis step, cracks marked with independent segments are actually an integrated crack. Unless those segments are connected, a full understanding of the crack is difficult. In this paper, a self-adaptive tensor voting algorithm is presented because the spatial proximity and the smoothness of the cracks are the main interfering factors. Thus, the crack fragments can be expressed in a tensor field that contains a ball tensor and a stick tensor. The two tensors can be defined as follows [26]:

Ball tensor: if point P is an isolated point, the tensor is expressed as $\left[\begin{array}{cc} 1 & 0\\ 0 & 1 \end{array}\right]$.

Stick tensor: if point P is a point on the curve, the tensor is expressed as $\left[\begin{array}{cc} \cos^{2}\theta & \sin\theta \cos\theta \\ \sin\theta \cos\theta & \sin^{2}\theta \end{array}\right]$. *θ* is the tangent angle between the tangent and the horizontal direction.

The stick tensor along the y-axis from the coordinate origin O and the voting strength from P can be calculated using the degradation function [27]:

$$\begin{array}{c} DF\left(s,k,\sigma \right)=e^{-\left(\frac{s^{2}+ck^{2}}{\sigma^{2}}\right)} \end{array}$$ (2)

where *σ* is the voting scale, $s=\frac{\theta l}{\sin\theta }$ is the arc length, $c=\frac{-16\log0.1\cdot (\sigma -1)}{\pi^{2}}$ controls the degradation speed of the function curvature, and $k=\frac{2\sin\theta }{l}$ is the curvature. *σ* is the free parameter of the voting field design; it directly controls the scale of the voting field. In previous studies, *σ* was usually set from prior knowledge; however, prior knowledge is difficult to obtain in underwater dam crack detection. Thus, parameter-adaptive fracture fragments are presented based on the clustering analysis. The scale of the voting field determines how much the neighborhood of the corresponding point impacts and also determines how much the neighborhood of the corresponding point is affected by its neighbors. As shown from the experiment, the number of marked cracks is inversely proportional to the voting field scale. Thus, the voting field scale is obtained as

$$\begin{array}{c} \sigma =k/n_{2} \end{array}$$ (3)

where k is the adjustment coefficient and *n*_2_ is the number of *c*_2_, which is obtained from the clustering step.

Then, a minimum spanning tree and edge pruning are used to further remove image noise and other false positives [28].

**Fig 4:**
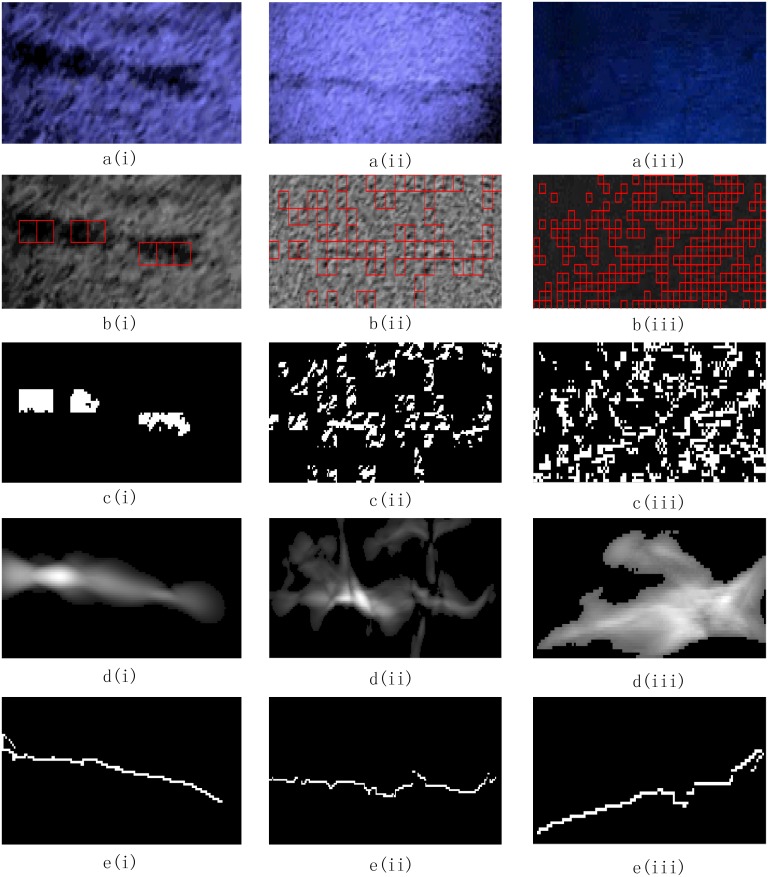
Image detection process results. a(i∼iii): original images, b(i∼iii): image blocks, c(i∼iii): crack fragments, d(i∼iii): crack probability map, e(i∼iii): final crack curves.

The proposed algorithm was used to analyze three typical cracks found in underwater dam surface sonar images captured at a hydropower dam project. And the sonar images taken at 1.8MHz were used to illustrate the detection algorithm process in detail. The first crack type is a large crack with a complex background (see a(i) in Fig 4); the second type is a medium crack (see a(ii) in Fig 4); and the third type is a tiny crack (see a(iii) in Fig 4). First, the three original sonar images were pre-processed; the results are shown in Fig 4(b). Then, the images were divided into blocks for clustering analysis, to mark the blocks that contains the crack information, as shown in Fig 4(c). Then, adaptive tensor voting was used to connect the marked crack fragments, as shown in Fig 4(d). Finally, the minimum spanning tree algorithm and edge pruning are used to obtain the marked cracks. The final crack shapes are shown in Fig 4(e).

### Underwater dam crack classification based on the fusion of images obtained from dual-frequency sonar

After obtaining the crack, a new crack classification algorithm based on the fusion of crack characteristics is put forward. There are three crack types: tiny cracks, medium cracks and large cracks. The tiny/medium/large cracks represent different severity levels of dam cracks. The division criteria of crack types is defined by the author’s research team according to the accurate measured dam crack of Longyangxia station. The crack classification are shown in Table 1. In this paper, the rare types of crack are not taking into account such as a crack is tiny in length but large in depth.

**Table 1:** Classification for different crack types.

The crack type	Length(mm)	Width(cm)	Depth(mm)
Tiny	<100	<2	<30
Medium	100 ∼ 500	2 ∼ 5	30 ∼ 100
Large	>500	>5	>100

The crack characteristics obtained from the images are used to classify the three crack types. There are several shape characteristics that can be used to describe a crack [29]. The relatively accurate features obtained from the detection part are the relative length (*C*) and the gray intensity ratio (*D*). The sonar images show that the relative length of cracks can accurately reflect their linear characteristics-slenderness. The grayscale intensity of cracks in an image reflects the fact that cracked region are darker than their surroundings. However, decision-making process based on *C* and *D* is different. Thus, a fuzzy expert rule base is established based on the statistical properties and experience. Here, the relative length and the gray intensity ratio obtained from the sonar images with high frequency patterns are denoted as *C*_1_ and *D*_1_. In addition, those with low frequency pattern features are labeled as *C*_2_ and *D*_2_.

In D-S theory, the total set of interested targets with mutually exclusive and exhaustive propositions is referred to as the frame of discernment (*FoD*), which is denoted as Θ = {*θ*_1_, *θ*_2_, ⋯, *θ*_*m*_}, where *θ*_*i*_ is the minimum identified level of information and *m* is the number of elements in the universal set. 2^Θ^ is used to denote the power set of Θ. In D-S theory, support for proposition *A* is provided via the basic belief assignment, which maps *m*_Θ_(.) : 2^Θ^ → [0, 1]. This mapping function satisfies

$$\begin{array}{c} m_{\Theta }\left(\phi \right)=0\;\text{and}\;\sum_{A\subseteq \Theta }m_{\Theta }\left(A\right)=1. \end{array}$$ (4)

Let Θ be the universal set representing all possible states under consideration. The pattern set of crack types is Θ = {*θ*_1_, *θ*_2_, *θ*_3_}, where *θ*_1_ represents tiny cracks, *θ*_2_ represents medium cracks, and *θ*_3_ represents large cracks. We observed that sonar used at 1.8MHz is sensitive to the relative lengths of cracks, providing one method for distinguishing between the three types of cracks. However, 1.8MHz sonar is insensitive to crack depth; therefore, it cannot be used to distinguish tiny cracks from medium cracks based solely on the gray intensity ratio. In contrast, sonar used at 1.1MHz is insensitive to crack relative length but it is sensitive to crack depth; thus, it provides another method for distinguishing between the three types of cracks.

**Fig 5:**
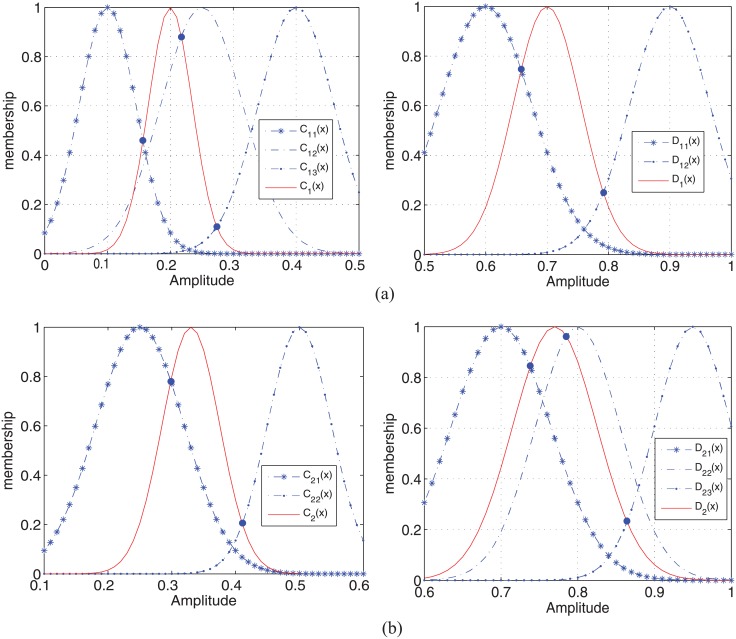
Statistical properties of sample characteristic set and test characteristics. (a) sonar images for 1.8 MHz pattern. (b) sonar images for 1.1 MHz pattern.

Based on these observations, three fuzzy partitions are established for *C*_1_ and *D*_2_. The fuzzy linguistic terms for *C*_1_ are S (Small amplitude), M (Medium amplitude), and H (Large amplitude). And the gaussian membership functions are constructed by calculating the mean value and the standard deviation value of relative length sample set which is obtained from each type of crack on the 1.8Hz sonar images. Similarly, the membership functions for *D*_1_, *C*_2_ and *D*_2_ are chosen by experiment statistics of sample characteristic set in the same way. The linguistic terms’ universe of *C*_1_ is *U*(*C*_1_) = (*C*_11_, *C*_12_, *C*_13_) and that of *D*_2_ is *U*(*D*_2_) = (*D*_21_, *C*_22_, *C*_23_), where the linguistic terms’ subscripts 1, 2, and 3 represent S, M, and H, respectively. In addition, two fuzzy partitions are established for *D*_1_ and *C*_2_, and the fuzzy linguistic term is S (Small amplitude) and H (Large amplitude). The linguistic terms’ universe of *C*_2_ is *U*(*C*_2_) = (*C*_21_, *C*_22_) and that of *D*_1_ is *U*(*D*_1_) = (*D*_11_, *D*_12_), where the linguistic terms’ subscripts 1 and 2 represent S and H. The membership function is shown in Fig 5.

In this paper, the BBAs are calculated using the likelihood measure *τ*(*C*_*k*_(*x*), *C*_*kq*_(*x*)), where *C*_*k*_(*x*) refers to the relative lengths of cracks. In addition, the likelihood measure *τ*(*C*_*k*_(*x*), *C*_*kq*_(*x*)) refers to the matching degree of *C*_*k*_(*x*) belonging to the linguistic terms *C*_*kq*_(*x*).

$$\begin{array}{c} \tau \left(C_{k},C_{kq}\right)={\text{sup}}_{x}\text{min}\left\{C_{k}\left(x\right),C_{kq}\left(x\right)\right\},\\ q=1,2,3;k=1\\ q=1,2;k=2. \end{array}$$ (5)

The matching degree is the maximum value of the intersecting point ordinate between the test membership degree curve and the sample membership degree curve. When the intersecting degree increases, the matching degree increases, which in turn provides greater evidence. The evidence reflects the extent of support, which is directly used as a basic belief assignment function after normalization. Thus, the BBA values can be calculated as follows:

$$\begin{array}{c} m_{\Theta }\left(C\right)\left[k\right]=\frac{\tau (C_{k},C_{kq})}{\sum_{k=1}^{n}\left[\tau \left(C_{k},C_{kq}\right)\right]}. \end{array}$$ (6)

Obviously, $\sum_{k=1}^{n}m_{\Theta_{O}}\left(C\right)\left[k\right]=1$. Similarly, the BBAs of the gray intensity are obtained as follows:

$$\begin{array}{c} m_{\Theta }\left(D)[k\right]=\frac{\tau (D_{k},D_{kq})}{\sum_{k=1}^{n}\left[\tau \left(D_{k},D_{kq}\right)\right]}. \end{array}$$ (7)

The likelihood measure *τ*(*D*_*k*_, *D*_*kq*_) can be calculated in the same way as *τ*(*C*_*k*_, *C*_*kq*_), where

$$\begin{array}{c} \tau \left(D_{k},D_{kq}\right)=\sup_{x}\min\left\{D_{k}\left(x\right),D_{kq}\left(x\right)\right\},\\ q=1,2;k=1\\ q=1,2,3;k=2. \end{array}$$ (8)

The previous item of the BBA can be calculated based on the Cartesian product stochastic relation, and the next item of the BBA can be calculated based on the edge random set’s expansion guidelines. The linguistic terms’ set of inputs *e* is *U*(*e*) = *U*_1_ × *U*_2_ × ⋯*U*_*n*_; here, *e* can be *C* or *D*. Thus, the random sets (*U*_*k*_, *m*_*kq*_), *q* = 1, ⋯, *J*_*k*_, are marginal random sets. In addition, (*U*, *M*_*In*_) can be obtained as the stochastic relation of a decomposable Cartesian product. Here,

$$\begin{array}{c} m_{In}\left(I\right)=m_{1}\left(I_{1q}\right)m_{2}\left(I_{2q}\right)\cdots m_{n}\left(I_{nq}\right), \end{array}$$ (9)

where *I* = (*I*_1*q*,_ *I*_2*q*,_⋯*I*_*nq*_) ∈ *U*, *q* ∈ {1, ⋯, *J*_*k*_}, *k* = 1, ⋯, *n*. Among the fuzzy rule bases, the relationship between the input and output can be expressed as $h=\bar{f}\left(I_{1q},I_{2q},\cdots \;I_{nq}\right)$; thus, the image of (*U*, *m*_*In*_) of the crack type space Θ is (ℜ, *m*_*Out*_), which can be obtained based on the random set extended criterion.

$$\begin{array}{c} ℜ=\{R_{j}=\bar{f}\left(I_{i}\right)|I_{i}\in U\} \end{array}$$ (10)

$$\begin{array}{c} m_{Out}\left(R_{j}\right)=\sum \left\{m_{In}\left(I_{i}\right)|R_{j}=\bar{f}\left(I_{i}\right)\right\}, \end{array}$$ (11)

where ℜ is the set class composed of the nonempty set of the Θ and *I*_*i*_ are the elements of *U*.

The mass function established using the matching degree of fuzzy features is the basic belief assignment of uncertainty. First, the two frequencies of the sonar images at arbitrary angles are used. Then, a fuzzy expert rule base is established based on the statistical properties and expert experience, as shown in Table 2.

**Table 2:** The fuzzy rules for BBA values of relative lengths and gray intensity ratios.

The number *α*	IF input		THEN output	IF input		THEN output
	m(C_1_)	m(D_1_)	Type	m(C_2_)	m(D_2_)	Type
1	C_11_	D_11_	*θ*_1_	C_21_	D_21_	*θ*_1_
2	C_11_	D_12_	*θ*_1_,*θ*_3_	C_21_	D_22_	*θ*_2_
3	C_12_	D_11_	*θ*_2_	C_21_	D_23_	Θ
4	C_12_	D_12_	*θ*_2_,*θ*_3_	C_22_	D_21_	*θ*_1_,*θ*_3_
5	C_13_	D_11_	Θ	C_22_	D_22_	*θ*_2_,*θ*_3_
6	C_13_	D_12_	*θ*_3_	C_22_	D_23_	*θ*_3_

To reduce the uncertainty of the classification and improve the robustness of the decision making, evidence from sonar imagery using both the high-frequency pattern and low-frequency pattern should be combined using the Dempster Combination Rule (DCR) [30]. Furthermore, this combination should increase the credibility of the classification, reducing the uncertainty to the maximum extent. Additional evidence for the two patterns from different perspectives can be obtained and combined in the same way. When new perspective evidence *B* is used to update the existing evidence *A*, the crack’s information becomes more comprehensive. In addition, conditional masses are used in this paper to update the evidence. The conditional masses can be calculated as follows [31]:

$$\begin{array}{c} m_{\Theta }\left(A\right)\left[k+1\right]=\alpha \left[k\right]m_{\Theta }\left(A\right)\left[k\right]+\sum_{B\subseteq \Theta }\beta (B)[k]m_{\Theta }\left(A|B\right)\left[k\right], \end{array}$$ (12)

where $\alpha \left[k\right]+\sum_{B\subseteq \Theta }\beta (B)[k]=1$, ∀*k* ≥ 0, and *β*(*B*)[⋅] = 0, ∀*B* ∉ ℑ_Θ_[⋅]. *m*_Θ_(*A*|*B*)[*k*] can be calculated as follows:

$$\begin{array}{c} m_{\Theta }\left(A|B\right)=\frac{\sum_{E:E\subseteq A}m(E)}{Pl\left(A\right)-\sum_{X:X\in l(A)}m(X)}-\sum_{E:E\subset A}m(E|B), \end{array}$$ (13)

where $l\left(A\right)=\left\{X\in \Theta :X=F\cup E,⌀\neq F\subseteq \bar{B,}⌀\neq E\subseteq A\subseteq B\right\}$.

## Results

**Fig 6:**
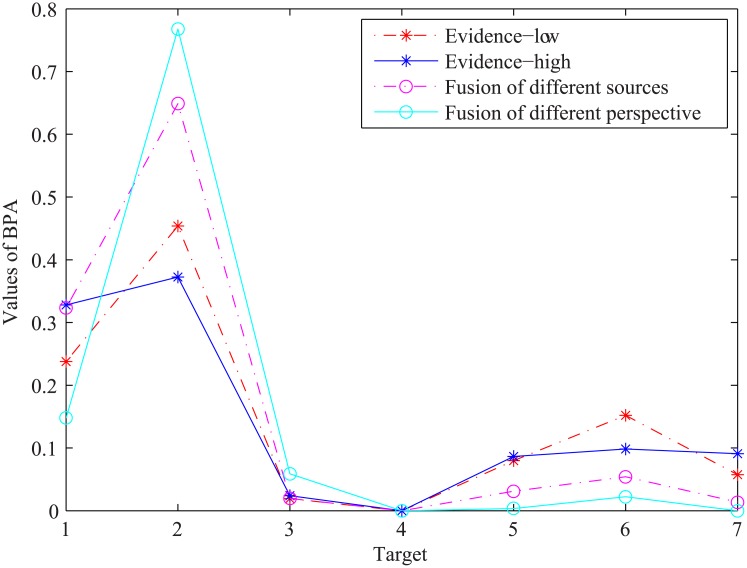
The fusion results from different perspectives alongside a comparison of different frequencies and their fusion.

To test the performance of the proposed approach, some experiments were performed. In this section, the results of the proposed algorithm for the 1.8 MHz and 1.1 MHz sonar images were given out. The BBA values of the relative lengths of the dam cracks in the sonar imagery can be obtained using formula (5) and (6). Similarly, the BBA values of the gray intensities are obtained using formula (7) and (8). Subsequently, the BBA values of the crack types are calculated using formula (9) and (11). To reduce the uncertainty of the classification, evidences from the two images at different frequencies are combined by DCR. The results are shown in Table 3 as evidence from one perspective. By rotating the sonar, 5 other pieces of evidence for the same area of dam surface from different perspectives are obtained in the same manner, and the results are shown in Table 4. Finally, evidence from all 6 perspectives is updated using formula (12) and (13). The results are shown in Fig 6.

**Table 3:** The BBA values obtained from sonar imagery using different frequencies and the results of the evidence fusion.

The Source of Evidence	The BBA values of the type of crack						
	m(*θ*_1_)	m(*θ*_2_)	m(*θ*_3_)	m(*θ*_1_*θ*_2_)	m(*θ*_1_*θ*_3_)	m(*θ*_2_*θ*_3_)	m(Θ)
1.1MHz	0.2378	0.4539	0.0192	0	0.0797	0.1520	0.0573
1.8MHz	0.3280	0.3725	0.0239	0	0.0865	0.0983	0.0908
fusion	0.3229	0.6488	0.0194	0	0.0311	0.0541	0.0137

**Table 4:** The BBA values for different perspectives.

The angle *α*	ε{*θ*_1_,*θ*_2_,*θ*_3_,*θ*_1_ *θ*_2_,*θ*_1_ *θ*_3_,*θ*_2_ *θ*_3_, Θ}						
	m(*θ*_1_)	m(*θ*_2_)	m(*θ*_3_)	m(*θ*_1_*θ*_2_)	m(*θ*_1_*θ*_3_)	m(*θ*_2_*θ*_3_)	m(Θ)
*α*_1_	0.3229	0.6488	0.0194	0	0.0311	0.0541	0.0137
*α*_2_	0.2142	0.5934	0.0308	0.0016	0.0298	0.0969	0.0333
*α*_3_	0.1033	0.5888	0.0096	0	0.0601	0.1241	0.1141
*α*_4_	0.1918	0.5934	0.0732	0.0911	0.0297	0.2297	0.0085
*α*_5_	0.1395	0.6114	0.0312	0	0.0669	0.0998	0.0512
*α*_6_	0.2318	0.6321	0.0315	0.0311	0.0297	0.0297	0.0141

## Discussion

**Fig 7:**
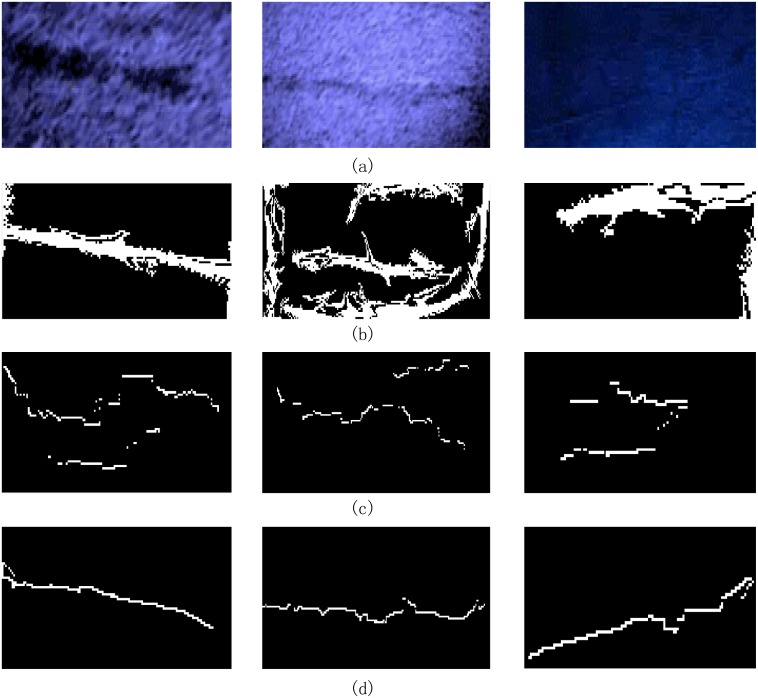
Image detection results comparing the proposed method and other classical methods. (a) Original image, (b) Tensor voting, (c) Wasp colony algorithm and (d) The proposed method.

To test the performance of the proposed approach for detection, the results are compared with those obtained using tensor voting [28] and the wasp colony algorithm [32]. The results are shown in Fig 7, in which the wasp colony algorithm is unable to effectively perform crack detection. The tensor voting method is able to detect the large cracks but could not detect small cracks. The results demonstrate that the proposed approach effectively solves the sonar crack detection problem.

The performance of the proposed approach for classification was tested using different frequencies and their fusion. The results show that the uncertainty of classification decreases substantially after the fusion of the different frequencies and perspectives. A total of 30 cracks for each type were conducted to test the proposed approach. The criteria of the classification is as follows: 1) The maximum BBA value should be greater than 0.65. 2) The m(Θ) should be less than 0.05. 3) The difference between the maximum BBA value and the other values should be greater than 0.2. The statistical results are shown in Table 5.

**Table 5:** Classification accuracy results for different crack types.

The crack type	1.8 MHz	1.1 MHz	Fusion of the two frequencies	Fusion of different perspectives
Tiny	46.7%	36.7%	76.7%	86.7%
Medium	63.3%	56.7%	83.3%	93.3%
Large	66.7%	76.7%	93.3%	93.3%

## Conclusions

This paper considered the underwater dam crack detection and classification problem, and proposed a novel approach. The statistical parameters of the image blocks constructed in the 3-D feature space and the image blocks are used to facilitate crack clustering analysis. Then, adaptive fracture fragments based on tensor voting are used to connect the crack fragments. The proposed crack detection algorithm can be applied to sonar images with low resolution, even though the cracks are tiny and subject to interference from other factors. The proposed crack classification algorithm can solve the underwater crack classification problem. In particular, when the test dam crack images and the sample images are both fuzzy, the proposed method still manages to obtain good performance. The experiments show that the proposed approach is able to effectively detect cracks and classify them accurately under complex underwater environments.

## Supporting information

S1 Fig A typical large crack sonar image. (BMP)

S2 Fig A typical medium crack sonar image. (BMP)

S3 Fig A typical tiny crack sonar image. (BMP)

S4 Fig The result of 3D clustering anylysis for the medium cack image. (BMP)

S5 Fig The result of 3D clustering for the large cack image. (BMP)

S6 Fig The result of 3D clustering for the tiny cack image. (BMP)

S7 Fig The tiny crack binary image after clustering. (BMP)

S8 Fig The medium crack binary image after clustering. (BMP)

S9 Fig The large crack binary image after clustering. (BMP)

S10 Fig The result of adaptive tensor voting for the large crack image. (BMP)

S11 Fig The result of adaptive tensor voting for the medium crack image. (BMP)

S12 Fig The result of adaptive tensor voting for the tiny crack image. (BMP)

S13 Fig The detection result for the large crack image. (BMP)

S14 Fig The detection result for the medium crack image. (BMP)

S15 Fig The detection result for the tiny crack image. (BMP)
