# Supplementary figures and images for: A novel underwater dam crack detection and classification approach based on sonar images
Source: PLoS One. 2017 Jun 22;12(6):e0179627. doi: 10.1371/journal.pone.0179627 (PMC5480977; doi:10.1371/journal.pone.0179627)

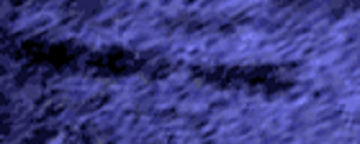

Supplement: S1 Fig — (BMP) [file pone.0179627.s001.bmp]

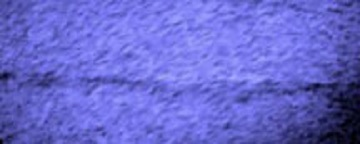

Supplement: S2 Fig — (BMP) [file pone.0179627.s002.bmp]

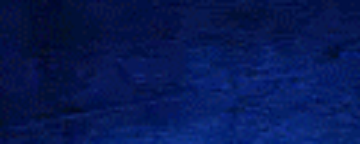

Supplement: S3 Fig — (BMP) [file pone.0179627.s003.bmp]

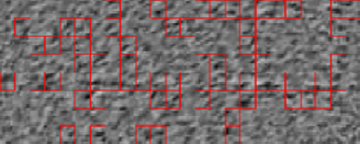

Supplement: S4 Fig — (BMP) [file pone.0179627.s004.bmp]

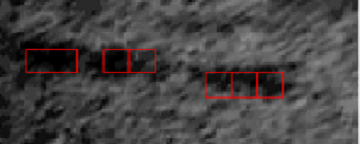

Supplement: S5 Fig — (BMP) [file pone.0179627.s005.bmp]

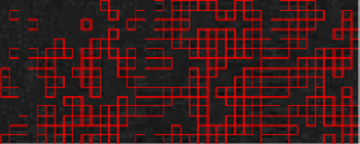

Supplement: S6 Fig — (BMP) [file pone.0179627.s006.bmp]

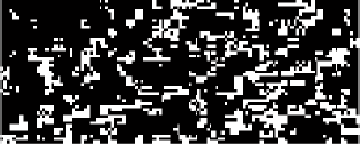

Supplement: S7 Fig — (BMP) [file pone.0179627.s007.bmp]

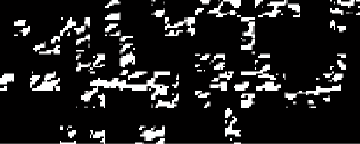

Supplement: S8 Fig — (BMP) [file pone.0179627.s008.bmp]

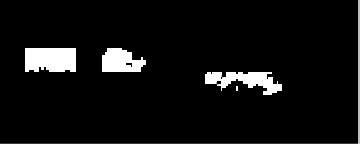

Supplement: S9 Fig — (BMP) [file pone.0179627.s009.bmp]

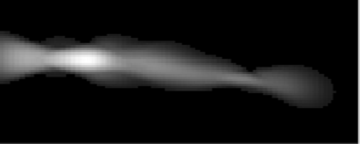

Supplement: S10 Fig — (BMP) [file pone.0179627.s010.bmp]

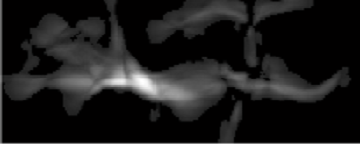

Supplement: S11 Fig — (BMP) [file pone.0179627.s011.bmp]

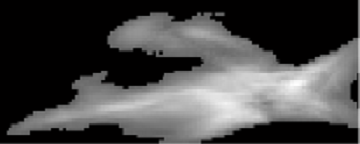

Supplement: S12 Fig — (BMP) [file pone.0179627.s012.bmp]

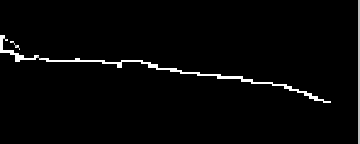

Supplement: S13 Fig — (BMP) [file pone.0179627.s013.bmp]

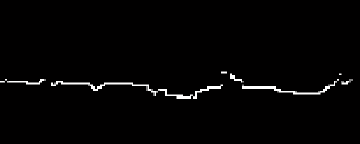

Supplement: S14 Fig — (BMP) [file pone.0179627.s014.bmp]

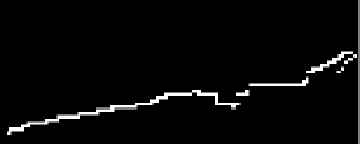

Supplement: S15 Fig — (BMP) [file pone.0179627.s015.bmp]
